# Evaluation of Antimicrobial Activity of Two Endodontic Sealers: Zinc Oxide with Thyme Oil and Zinc Oxide Eugenol against Root Canal Microorganisms— An *in vitro* Study

**DOI:** 10.5005/jp-journals-10005-1489

**Published:** 2018-04-01

**Authors:** Nilima R Thosar, Manoj Chandak, Manohar Bhat, Silpi Basak

**Affiliations:** 1 Professor, Department of Pedodontics and Preventive Dentistry, Sharad Pawar Dental College & Hospital, Datta Meghe Institute of Medical Sciences, Wardha, Maharashtra, India; 2 Professor and Head, Department of Conservative Dentistry and Endodontics, Sharad Pawar Dental College & Hospital, Datta Meghe Institute of Medical Sciences, Wardha, Maharashtra, India; 3 Professor and Head, Department of Pedodontics and Preventive Dentistry, Jaipur Dental College, Jaipur, Rajasthan, India; 4 Professor, Department of Microbiology, Jawaharlal Nehru Medical College Datta Meghe Institute of Medical Sciences, Wferdha, Maharashtra India

**Keywords:** Antimicrobial efficacy, Zinc oxide eugenol paste, Zinc oxide mixed with thyme oil paste.

## Abstract

**Aim:**

The present study was aimed to find out and compare the antimicrobial effect of the paste containing zinc oxide cement mixed with thyme oil (ZO + Th oil) with that of the paste containing zinc oxide and eugenol (ZO + E) against *Staphylococcus aureus, Escherichia coli, Enterococcus faecalis,* and *Pseudomonas aeruginosa,* common root canal pathogens of deciduous teeth.

**Materials and methods:**

An *in vitro* antimicrobial effect was carried out by the agar diffusion method. The ZO + Th oil paste was filled in the punched holes of Mueller Hinton agar at two equidistant points. The same was followed for ZO + E paste. For 24 hours, all the plates were incubated at a temperature of 37°C. The inhibition zones in millimeters around the wells were calculated. There were 6 times repetitions of the test for each microorganism. Data were tabulated and analyzed statistically using one-way analysis of variance (ANOVA) and Tukey’s *post hoc* comparison test. Level of significance for the tests was 5%.

**Results:**

Zones of bacterial inhibition were highest for ZO + Th oil paste against the pathogenic microorganisms **S.** *aureus,* followed by *E. coli, E. faecalis, P. aeruginosa* while for ZO + E paste, the decreasing order against pathogenic microorganisms was *E. coli, S. aureus, E. faecalis,* and *P. aeruginosa.* Statistically significant difference was found in both the pastes, ZO + Th oil paste and ZO + E paste.

**Conclusion:**

ZO + Th oil paste showed higher levels of antimicrobial effect against the root canal pathogens.

**How to cite this article:** Thosar NR, Chandak M, Bhat M, Basak S. Evaluation of Antimicrobial Activity of Two Endodontic Sealers: Zinc Oxide with Thyme Oil and Zinc Oxide Eugenol against Root Canal Microorganisms—An *in vitro* Study. Int J Clin Pediatr Dent 2018;11(2):79-82.

## INTRODUCTION

Microbial infections in root canals of primary teeth are polymicrobial in nature.^1^ Pulpectomy of primary teeth includes biomechanical preparation and use of intracanal dressing with antibacterial properties. The success of endodontic treatment depends on the removal of infected bacteria.^2^ There are various methods: proper debridement with instrumentation, antibacterial irrigations, and antibacterial filling materials.^3^

Due to the presence of numerous accessory canals in primary teeth, intracanal dressings may fail to remove these microorganisms from inaccessible areas. So, it is important to use an obturating material which has antibacterial properties to act on such microorganisms of inaccessible areas of root canals of primary teeth. Due to antibacterial efficacy, thyme oil shows its potential to be used in dentistry.^4^

Thymol inhibits the bacterial growth in oral cavity. It shows potential to inhibit the dental infection also.^5,6^ Thymol and carvacrol are its important constituents in 20 to 40%. Others include B-cymene, pinene and triterpenic acid, menthone, borneol, linalool, and cineole.^7^ As there are no studies available in the literature about the use of thyme oil as obturating material in deciduous teeth, the present study was carried out by using ZO + Th oil paste and compared with ZO + E paste.

Therefore, the present study was aimed to find out the antimicrobial activity of ZO + Th oil paste with that of ZO + E paste.

## MATERIALS AND METHODS

The present study was an *in vitro* study. It was approved by the institutional ethical committee of Datta Meghe Institute of Medical Sciences. In this study, zinc oxide was combined with thyme oil. It was compared with zinc oxide and eugenol combination paste. Thyme oil was procured from Aromatantra, Mumbai.

The powder-liquid ratio of pastes was as per specifications of Tchaou et al.^3^ A quantity of powder 0.2 gm was mixed with 0.07 cc oil. Mixing of powder with liquid was carried out with the help of spatula on a dry glass slab.^8^ Microorganisms from the microbiology department of Jawaharlal Nehru Medical College, Wardha, were used for the study.

Microbial strains studied were: *S. aureus* [American Type Culture Collection (ATCC) 25923], *E. faecalis* (ATCC 29212), *E. coli* (ATCC 25922), *P. aeruginosa* (ATCC 27853). Mueller Hinton agar was used as the growth medium for testing the susceptibility of *S. aureus, E. coli, Efaecalis,* and *P aeruginosa.^9^*

Stock culture of test microorganism was poured in brain heart infusion broth (5 mL). Incubation was done at 37°C for 24 hours. Microorganisms were then subcul-tured on blood agar; again incubated for the same time and same temperature. Colonies of microorganisms were inoculated in the medium of nutrient broth for the time of 6 hours. Its density was adjusted to 0.5 as per McFarland scale.^10^ Petri dishes (90 mm) with 4 mm thick Mueller Hinton agar was used. All the work was done under laminar air flow chamber.

The lawn technique was used for uniform distribution of bacterial dilutions. Care was taken while punching the holes in agar; the mean diameter of holes was 6 mm at the level of equal distance from each other. Holes were punched with open end of micropipette. Freshly prepared root canal filling paste was then placed in the hole. It was kept at room temperature for 2 hours.

**Table 1:** **Table 1:** Zones of bacterial growth inhibition in mm of ZO + Th oil against four bacterial strains

										*95% confidence interval for mean*							
*Material*		*n*		*Mean*		*Std. deviation*		*Std. error*		*Lower bound*		*Upper bound*		*Minimum*		*Maximum*	
*S. aureus*		6		36.33		1.36		0.55		34.89		37.76		35.00		38.00	
*E. coli*		6		35.33		2.73		1.11		32.46		38.20		32.00		38.00	
*E. faecalis*		6		28.00		1.78		0.73		26.12		29.87		26.00		30.00	
*P. aeruginosa*		6		21.33		7.22		2.95		13.74		28.92		14.00		30.00	

The procedure was repeated for 6 times for every microorganism. The plates were incubated for 24 hours. Next day, the diameter of growth inhibitory zones was calculated by using the antibiotic zone scale of HiMedia.^8^ Wider zones were read as having a higher antibacterial effect against the specific microorganism.^9^

Data were analyzed statistically using ANOVA and Tukey’s *post hoc* test at a significance level of 5% using the GraphPad Prism 4 software.

## RESULTS

For ZO + Th oil paste, the diameter in mm of the inhibition zones for *S. aureus* was 36.33 ± 1.36, for *E. coli,* it was 35.33 ± 2.73, for *E. faecalis,* it was 28 ± 1.78, and for *P. aeruginosa,* it was 21.33 ± 7.22 (Table 1).

Table 2 is suggestive of the one-way ANOVA of bacterial inhibition zones for the ZO + Th oil paste group in which the difference between and within groups was statistically significant (p-value: 0.0001, p < 0.05).

Table 3 shows multiple comparisons: Tukey test was done for the ZO + Th oil paste group to evaluate the inhibitory zones of bacteria. It showed that the difference was statistically significant for *S. aureus* and *E. faecalis* with a p-value 0.004, which is less than 0.05; however, significant differences were also found for other bacteria also, i.e., between *S. aureus* and *P. aeruginosa* with p-value 0.0001; between *E. coli* and *E. faecalis* (0.013, p < 0.05); between *E. coli* and *P. aeruginosa,* 0.0001; and between *E. faecalis* and *P. aeruginosa* (p-value: 0.027, p < 0.05).

**Table 2:** **Table 2:** One-way ANOVA of zones of bacterial growth inhibition of ZO + Th oil against four bacterial strains

*Source of variation*		*Sum of squares*		*Df*		*Mean square*		*f-value*		*p-value*	
Between groups		2756.80		4		689.20		53.17		0.0001 S,	
Within groups		324.00		25		12.96				p < 0.05	
Total		3080.80		29							

S: Significant

**Table 3:** **Table 3:** Multiple comparison: Tukey test of zones of bacterial growth inhibition of ZO + Th oil against four bacterial strains

										*95% confidence interval*			
*Microorganisms*				*Mean difference*		*Std. error*		*p-value*		*Lower bound*		*Upper bound*	
*S. aureus*		*E. coli*		1.00		2.07		0.988, NS		–5.10		7.10	
		*E. faecalis*		8.33		2.07		0.004, S		2.22		14.43	
		*P. aeruginosa*		15.00		2.07		0.0001, S		8.89		21.10	
*E. coli*		*E. faecalis*		7.33		2.07		0.013, S		1.22		13.43	
		*P. aeruginosa*		14.00		2.07		0.0001, S		7.89		20.10	
*E. faecalis*		*P. aeruginosa*		6.66		2.07		0.027, S		0.56		12.77	

NS: Not significant; S: Significant

**Table 4:** **Table 4:** Zones of bacterial growth inhibition in mm of ZOE against four bacterial strains

										*95% confidence interval for mean*							
*Microorganisms*		*n*		*Mean*		*Std. deviation*		*Std. error*		*Lower bound*		*Upper bound*		*Minimum*		*Maximum*	
*S. aureus*		6		16.00		0.00		0.00		16.00		16.00		16.00		16.00	
*E. coli*		6		19.00		1.09		0.44		17.85		20.14		18.00		20.00	
*E. faecalis*		6		10.83		1.47		0.60		9.28		12.37		9.00		12.00	
*P. aeruginosa*		6		10.33		0.51		0.21		9.79		10.87		10.00		11.00	

**Table 5:** **Table 5:** One-way ANOVA of zones of bacterial growth inhibition of ZOE against four bacterial strains

*Source of variation*		*Sum of squares*		*Df*		*Mean square*		*f-value*		*p-value*	
Between groups		352.20		4		88.05		95.70		0.0001 S,	
Within groups		23.00		25		0.92				p < 0.05	
Total		375.20		29							

S: Significant

The difference for the inhibitory zones between *S. aureus* and *E. coli* (0.988, p < 0.05) was not significant statistically. Diameters in mm of the inhibitory zones for other microorganisms in the ZO + E paste group in descending order were: *E. coli* (19 ± 1.09), *S. aureus* (16 ± 0.00), *E. faecalis* (10.83 ± 1.47), and *P. aeruginosa* (10.33 ± 0.51) (Table 4) respectively.

In Table 5, one-way ANOVA for the ZO + E paste group, differences for inhibitory zones of bacteria are found to be statistically significant (p-value: 0.0001, p < 0.05) between and within groups. Table 6 shows multiple comparisons: Tukey test for inhibitory zones of bacteria and it was observed that the difference between *S. aureus* and *E. coli* was found to be statistically significant (0.0001, p < 0.05). For other microorganism, the difference was found to be statistically significant like *S. aureus vs E. faecalis* (0.0001, p < 0.05); *S. aureus vs P. aeruginosa* (0.0001, p < 0.05); *E. coli vs E. faecalis* (0.0001, p < 0.05); and *E. coli* *vs E. faecalis* (0.002, p < 0.05).

The difference for *E. faecalis vs P. aeruginosa* (0.893, p < 0.05) was not statistically significant.

**Table 6:** **Table 6:** Multiple comparison: Tukey test of zones of bacterial growth inhibition of ZOE against four bacterial strains

										*95% confidence interval*			
*Microorganisms*				*Mean difference*		*Std. error*		*p-value*		*Lower bound*		*Upper bound*	
*S. aureus*		*E. coli*		–3.00000		0.55377		0.0001, S		–4.62		–1.37	
		*E. faecalis*		5.16667		0.55377		0.0001, S		3.54		6.79	
		*P. aeruginosa*		5.66667		0.55377		0.0001, S		4.04		7.29	
*E. coli*		*E. faecalis*		8.16667		0.55377		0.0001, S		6.54		9.79	
		*P. aeruginosa*		8.66667		0.55377		0.0001, S		7.04		10.29	
*E. faecalis*		*P. aeruginosa*		0.50000		0.55377		0.893, NS		–1.12		2.12	

NS: Not significant; S: Significant

## DISCUSSION

Infections of root canals in primary teeth are polymicro-bial in nature. Numerous materials have been tried in dentistry for obturation of deciduous teeth. Zinc oxide is widely used in dentistry.

There are disadvantages associated with this material like slow resorption of ZOE as compared with physiologic resorption of deciduous tooth, deflection of succedaneous tooth, irritation to tissue at periapical area of tooth, bone and cementum necrosis and tooth discoloration.^11^ Thyme oil is found in European countries around Mediterranean and said to belong to around 300 species of shrubs and plants.

Essential oil from thyme plant is prepared from its leaves and flowers by the method of steam distillation. Thyme oil is useful in certain conditions like relief from gastritis and enterocolitis. It is also found to be useful in oral thrush. It can be used for patients suffering from asthma and respiratory infections. Other uses of it are treatment of swelling caused by gout or rheumatic problems, for backache, joint pains, and sciatica.

Thyme oil is also used for other conditions like vaginitis, urinary infections, etc.^7^ Very few review articles are available in the literature mentioning the use of thyme oil in dentistry. Antibacterial effects of thymol have been found in Listerine.^12^ The study of Skold-Larsson et al^13^ had shown the use of thymol in the form of dental varnish to reduce the *Streptococcus mutans* levels in supragingival plaque near the bracket in patients with orthodontic brackets. Thyme oil and clove oil together had shown the antimicrobial effect against *E. coli, S. aureus,* and *C. albicans* at various concentrations of the extracts.^14-16^ Thosar et al^17^ in their study carried out in 2013 had shown the antimicrobial susceptibility for thyme oil for *E. coli* which was with minimum inhibitory concentration (MIC): 2 μL/mL, minimum bactericidal concentration (MBC): 8 μL/mL; *for C. albicans,* MIC, MBC: 16 μL/mL; for *E. faecalis* MIC, MBC: 32 μL/mL and for *S. aureus* MIC, MBC: 32 μL/mL respectively. The present study suggests that use of thyme oil when mixed with zinc oxide powder in the form of paste showed wider zones of inhibition *for S. aureus, E. faecalis, E. coli,* and *P. aeruginosa* in comparison with zinc oxide powder which was mixed with eugenol oil.

As the ZO + Th oil paste showed more antibacterial activity in comparison with ZOE paste, it can be successfully used in pediatric dentistry as an obturating material in primary teeth. But further elaborative animal experimental studies can prove its tissue biocompatibility and toxicity properties.

## CONCLUSION

A new material, i.e., ZO + Th oil paste, used in this study had shown strong antibacterial activity against all the root canal microorganisms which were studied and showed its superiority over the zinc oxide + eugenol paste group.

This material will definitely prove its success in pediatric dentistry as an obturating material for primary teeth.
